# Supplementary material for: Molecular-level insight into photocatalytic CO2 reduction with H2O over Au nanoparticles by interband transitions
Source: Nat Commun. 2022 Jul 6;13:3894. doi: 10.1038/s41467-022-31474-2 (PMC9259601; doi:10.1038/s41467-022-31474-2)
Supplement: Supplementary file 1 — Supplementary Information [file 41467_2022_31474_MOESM1_ESM.pdf]

Supporting Information

Molecular-level insight into photocatalytic CO_2 reduction with H_2O over Au nanoparticles by interband transitions

Wenchao Shangguan[1], Qing Liu[1], Ying Wang[2,*], Ning Sun[1], Yu Liu[3], Rui Zhao[3], Yingxuan Li[1,*], Chuanyi Wang[1], and Jincai Zhao[4]

1. School of Environmental Science and Engineering, Shaanxi University of Science and Technology, Xi'an 710021, China.

2. State Key Laboratory of Rare Earth Resource Utilization, Changchun Institute of Applied Chemistry, Chinese Academy of Sciences, Changchun 130022, China.

3. Engineering Research Center of Advanced Functional Material Manufacturing of Ministry of Education, School of Chemical Engineering, Zhengzhou University, Zhengzhou 450001, China

4. Key Laboratory of Photochemistry, CAS Research/Education Center for Excellence in Molecular Sciences, Institute of Chemistry, Chinese Academy of Sciences, Beijing 100190, P. R. China.

*To whom correspondence should be addressed. E-mail: Y.L. (email: liyingxuan@sust.edu.cn); Y.W. (email: ywang_2012@ciac.ac.cn).

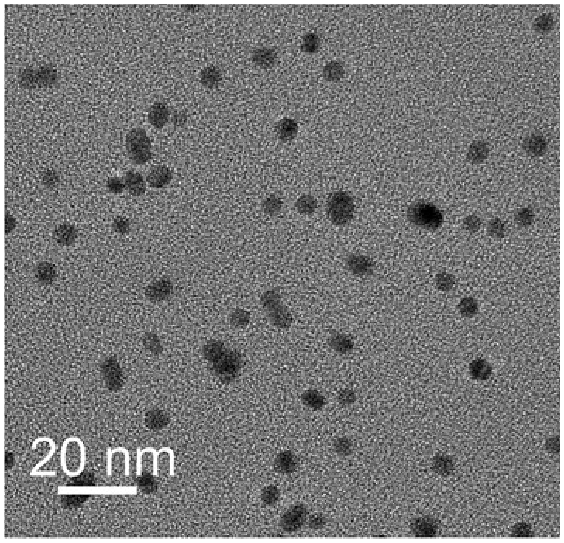

Supplementary Fig. 1 | TEM image. The enlarged TEM image of Au NPs.

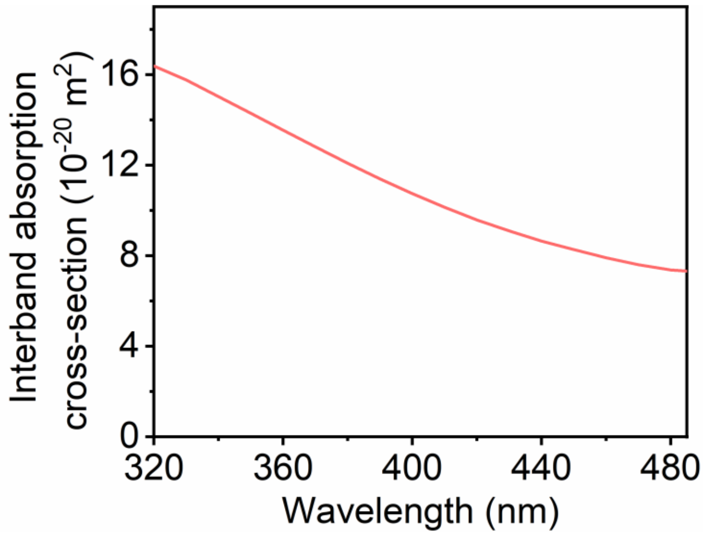

Supplementary Fig. 2 | Light absorption cross-section. Calculated wavelength-dependent interband absorption cross-section of 4 nm Au NPs.

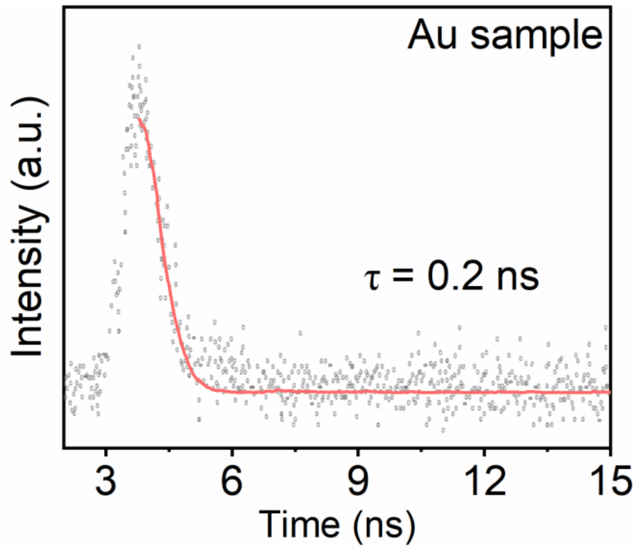

Supplementary Fig. 3 | Lifetime of the photoinduced charge carriers. TRPL decay spectrum of Au NPs.

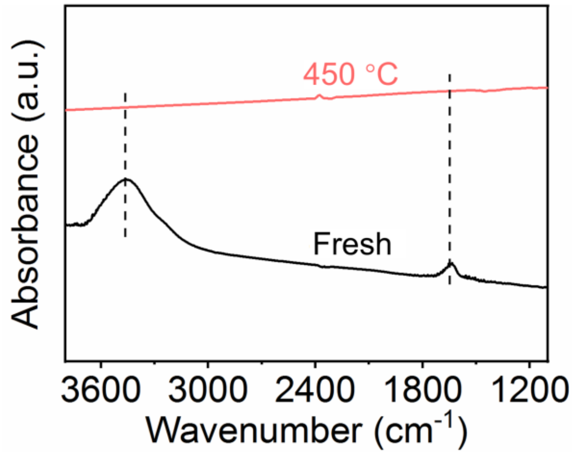

Supplementary Fig. 4 | FT-IR spectra. FT-IR spectra of fresh Au sample (black curve) and the Au sample treated at 450 °C for 1 h under flowing argon atmosphere (red curve).

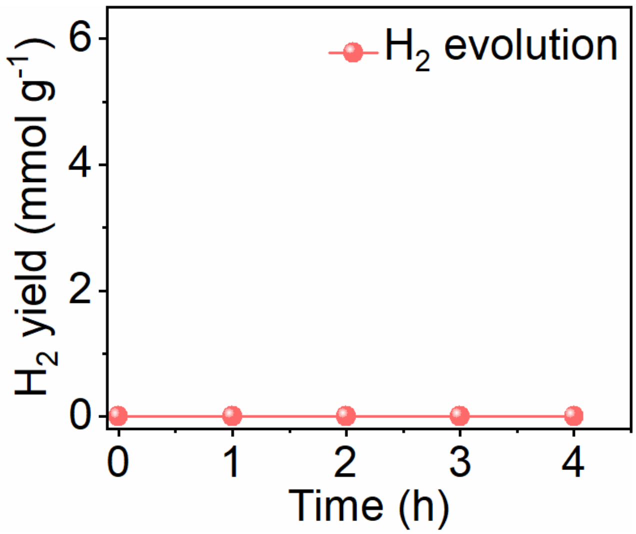

Supplementary Fig. 5 | Photocatalytic H_2 evolution. H_2 production on Au NPs under 420 nm LED light irradiation at 200 °C.

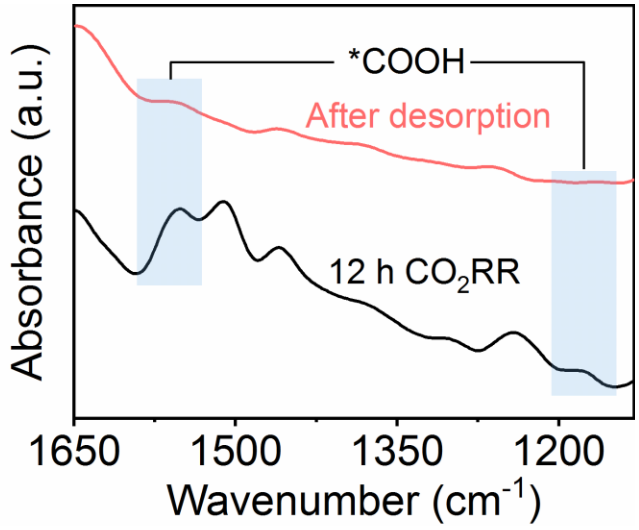

Supplementary Fig. 6 | FT-IR spectra. FT-IR spectra of the Au catalysts before (12 h reaction) and after desorption treatment.

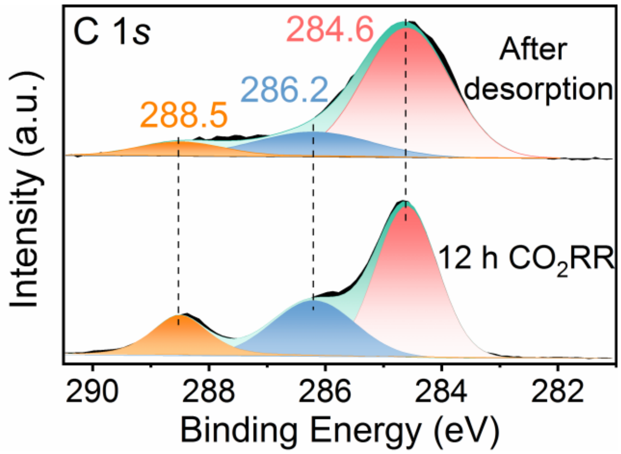

Supplementary Fig. 7 | XPS spectra. High-resolution C 1*s* XPS spectra of the Au catalysts before (12 h reaction) and after desorption treatment.

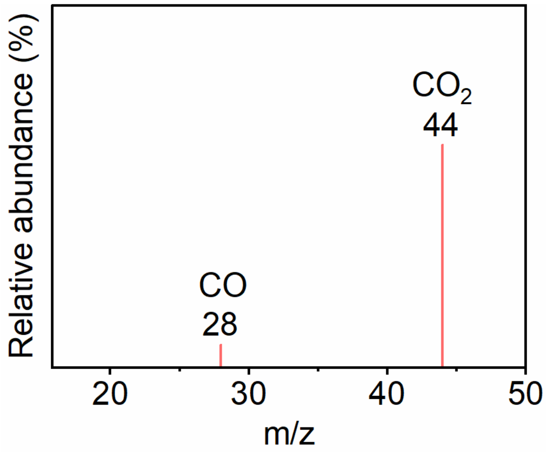

Supplementary Fig. 8 | Mass spectra. Mass spectrum from GC-MS analysis of CO generated from photocatalytic CO_2RR with H_2 over Au NPs.

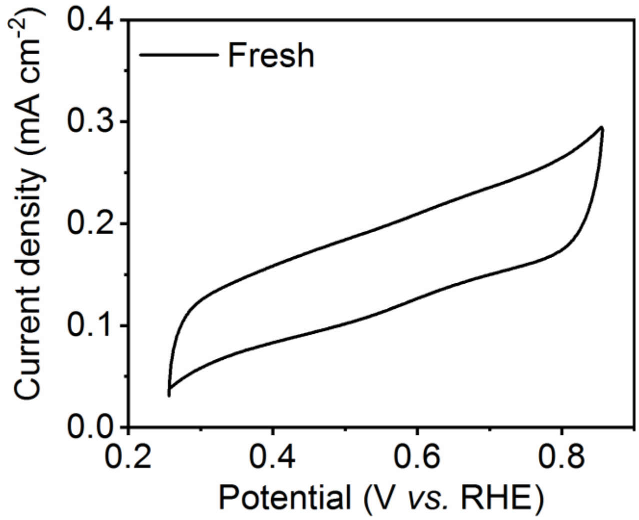

Supplementary Fig. 9 | Electrochemical characterization. CV curve of fresh Au.

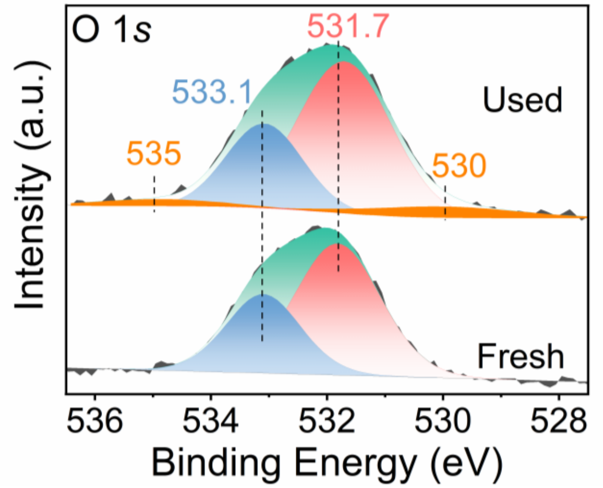

Supplementary Fig. 10 | XPS spectra. High-resolution O 1*s* spectra of fresh and used (3 h reaction) Au NPs.

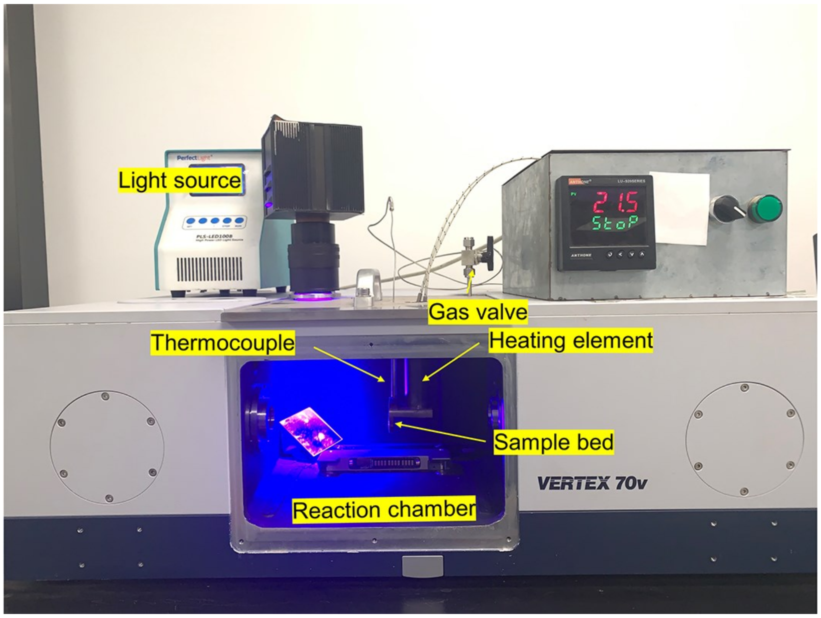

Supplementary Fig. 11 | The instrument for *in situ* FT-IR tests. Photo of the instrument used for *in situ* FT-IR measurements.

Supplementary Table 1. Light intensity corresponding to different wavelengths of monochromatic LED light sources. The distance from the light source to the intensity meter is 13.5 cm, which is consistent with the distance of the sample. The values of light intensity presented are the maximum values after adjustment.

Light source	Light intensity (mW cm^{-2})
365 nm	48
420 nm	73
450 nm	94
520 nm	33
590 nm	23
620 nm	59

Supplementary Table 2. Summary of some representative semiconductor based photocatalysts for CO_2 reduction with corresponding phase compositions, reaction conditions and catalytic activity.

Photocatalyst	Reaction conditios	Catalytic activity	Ref.
In-doped TiO_2	$CO_2 + H_2O$<br>373K<br>500 W Hg lamp	243.7 μmol g^{-1} h^{-1} CO<br>81.2 μmol g^{-1} h^{-1} CH_4	1
V_{Bi}-BiOBr	$CO_2 + H_2O$<br>Room temperature<br>300 W Xe lamp	20.1 μmol g^{-1} h^{-1} CO	2
Boron	$CO_2 + H_2O$<br>> 300 °C<br>300 W Xe lamp	1.0 μmol h^{-1} CO<br>2.5 μmol h^{-1} CH_4	3
Cu_0/Cu_2O	$CO_2 + H_2O$<br>383K<br>300 W Xe lamp<br>(> 400 nm, 400 mW cm^{-2})	13.2 μmol g^{-1} h^{-1} CO<br>2.6 μmol g^{-1} h^{-1}<br>CH_3OH	4
Au/p-GaN	$CO_2 + H_2O$<br>AM1.5 G	150 μmol g^{-1} h^{-1} CO	5
Cu−Bi/$BiVO_4$	$CO_2 + H_2O$<br>Xe lamp<br>(160 mW cm^{-2})	11.15 μmol g^{-1} h^{-1} CO<br>1.54 μmol g^{-1} h^{-1} CH_4	6
Bi_2S_3/UIO-66	$CO_2 + H_2O$<br>150 °C<br>Full−spectrum light<br>(650 mW cm^{-2})	25.6 μmol g^{-1} h^{-1} CO	7
(Au/A-TiO_2)@g-C_3N_4	$CO_2 + H_2O$<br>300 W Xe lamp<br>(λ ≥ 420 nm, 80 mW cm^{-2})	21.7 μmol g^{-1} h^{-1} CO<br>37.4 μmol g^{-1} h^{-1} CH_4	8
V_O-BiOBr atomic layers	$CO_2 + H_2O$<br>300 W Xe lamp<br>(λ > 400 nm)	87.4 μmol g^{-1} h^{-1} CO	9

$Bi_4Ti_3O_{12}$	CO_2 + H_2O<br>25 °C<br>300 W Xe lamp	15.1 μmol g^{-1} h^{-1} CO	10
Au-TiO_2 (tetragonal bipyramids)	CO_2 + H_2O<br>300 W Xe lamp<br>(UV light irradiation)	25.9 μmol g^{-1} h^{-1} CO<br>5.3 μmol g^{-1} h^{-1} CH_4	11
Co_2N/BiOBr	CO_2 + H_2O<br>300 W Xe lamp	67.8 μmol g^{-1} h^{-1} CO<br>31.7 μmol g^{-1} h^{-1} O_2	12
Au-Mesoporous TiO_2 Nanospheres	CO_2 + H_2O<br>180 °C<br>Full−spectrum<br>(0.72 W cm^{-2},)	3.36 μmol g^{-1} h^{-1} CO<br>5.0 μmol g^{-1} h^{-1} CH_4	13
CN/$Bi_9O_{7.5}S_6$ (2D/2D Van der Waals heterojunction)	CO_2 + H_2O<br>300 W Xe lamp<br>(λ > 420 nm)	18.6 μmol g^{-1} h^{-1} CO<br>4.17 μmol g^{-1} h^{-1} O_2	14
Pt/BP-Bi_2WO_6-OVs	CO_2 + H_2O + CH_3CN + TEOA<br>300 W Xe lamp<br>(250 mW cm^{-2})	20.5 μmol g^{-1} h^{-1} CO<br>16.8 μmol g^{-1} h^{-1} H_2	15
Au NPs	CO_2 + H_2O<br>200 °C<br>LED−420 nm<br>(73 mW cm^{-2})	4.73 mmol g^{-1} h^{-1} CO<br>1.98 mmol g^{-1} h^{-1} O_2	**This work**

Supplementary References

1. Tahir, M. & Amin, N. S. Indium-doped TiO_2 nanoparticles for photocatalytic CO_2 reduction with H_2O vapors to CH_4. *Appl. Catal., B* **162**, 98−109 (2015).
2. Di, J. et al. Bismuth vacancy-tuned bismuth oxybromide ultrathin nanosheets toward photocatalytic CO_2 reduction. *ACS Appl. Mater. Interfaces* **11**, 30786−30792 (2019).
3. Liu, G. et al. Elemental boron for efficient carbon dioxide reduction under light irradiation. *Angew. Chem., Int. Ed.* **56**, 5570−5574 (2017).
4. Zheng, Y. et al. Controlled synthesis of Cu_0/Cu_2O for efficient photothermal catalytic conversion of CO_2 and H_2O. *ACS Sustainable Chem. Eng.* **9**, 1754−1761 (2021).
5. Li, R. et al. Unassisted highly selective gas-phase CO_2 reduction with a plasmonic Au/p-GaN photocatalyst using H_2O as an electron donor. *ACS Energy Lett.* **6**, 1849−1856 (2021).
6. Huang, L., Duan, Z., Song, Y., Li, Q. & Chen, L. $BiVO_4$ microplates with oxygen vacancies decorated with metallic Cu and Bi nanoparticles for CO_2 photoreduction. *ACS Appl. Nano Mater.* **4**, 3576−3585 (2021).
7. Chen, X., Li, Q., Li, J., Chen, J. & Jia, H. Modulating charge separation via in situ hydrothermal assembly of low content Bi_2S_3 into UiO-66 for efficient photothermocatalytic CO_2 reduction. *Appl. Catal., B* **270**, 118915 (2020).
8. Wang, C. et al. Efficient Z-scheme photocatalysts of ultrathin g-C_3N_4-wrapped Au/TiO_2-nanocrystals for enhanced visible-light-driven conversion of CO_2 with H_2O. *Appl. Catal., B* **263**, 118314 (2020).

9. Wu, J. et al. Efficient visible-light-driven CO_2 reduction mediated by defect-engineered BiOBr atomic layers. *Angew. Chem., Int. Ed.* **57**, 8719−8723 (2018).

10. Liu, L. et al. Synergistic polarization engineering on bulk and surface for boosting CO_2 photoreduction. *Angew. Chem., Int. Ed.* **60**, 18303−18308 (2021).

11. Wang, A. et al. Interfacial facet engineering on the schottky barrier between plasmonic Au and TiO_2 in boosting the photocatalytic CO_2 reduction under ultraviolet and visible light irradiation. *Chem. Eng. J.* **404**, 127145 (2021).

12. Di, J. et al. Cobalt nitride as a novel cocatalyst to boost photocatalytic CO_2 reduction. *Nano Energy* **79**, 105429 (2021).

13. Cai, S., Chen, J., Li, Q. & Jia, H. Enhanced photocatalytic CO_2 reduction with photothermal effect by cooperative effect of oxygen vacancy and Au cocatalyst. *ACS Appl. Mater. Interfaces* **13**, 14221−14229 (2021).

14. Li, J. et al. Van der Waals heterojunction for selective visible-light-driven photocatalytic CO_2 reduction. *Appl. Catal., B* **284**, 119733 (2021).

15. Chen, C. et al. Ambient-stable black phosphorus-based 2D/2D S-scheme heterojunction for efficient photocatalytic CO_2 reduction to syngas. *ACS Appl. Mater. Interfaces* **13**, 20162−20173 (2021).
